# Obsessive–compulsive disorder patients have a reduced sense of control on the illusion of control task

**DOI:** 10.3389/fpsyg.2014.00204

**Published:** 2014-03-13

**Authors:** Claire M. Gillan, Sharon Morein-Zamir, Alice M. S. Durieux, Naomi A. Fineberg, Barbara J. Sahakian, Trevor W. Robbins

**Affiliations:** ^1^Department of Psychology, New York UniversityNew York, NY, USA; ^2^Behavioural and Clinical Neuroscience Institute, University of CambridgeCambridge, UK; ^3^Department of Psychology, University of CambridgeCambridge, UK; ^4^Department of Psychiatry, University of CambridgeCambridge, UK; ^5^Institute of Psychiatry, Kings College LondonLondon, UK; ^6^Department of Psychiatry, Queen Elizabeth II HospitalHertfordshire, UK; ^7^Postgraduate School of Medicine, University of HertfordshireHatfield, UK

**Keywords:** OCD, control, illusion of control, compulsivity, anxiety disorders

## Abstract

There is disagreement regarding the role of perceived control in obsessive–compulsive disorder (OCD). The present study used a traditional illusion of control paradigm (Alloy and Abramson, 1979) to empirically test control estimation in OCD. Twenty-six OCD patients and 26 matched comparison subjects completed an illusion of control task wherein their goal was to attempt to exert control over a light bulb. The density of reinforcement (high, low) and the valence of trials (gain, loss) were experimentally manipulated within subjects. Unbeknownst to participants, the illumination of the light bulb was predetermined and irrespective of their behavior. OCD patients exhibited lower estimates of control compared with healthy comparison subjects. There were no interactions between group and outcome density or group and valence. We found that OCD patients endorse lower estimates of control than comparison subjects. This finding highlights a potential role for contingency learning in the disorder.

## INTRODUCTION

Obsessive–compulsive disorder (OCD) is a condition in which patients suffer from distressing obsessive thoughts (e.g., that harm will come to a loved one) and feel compelled to perform actions (e.g., repeatedly counting or checking; APA, 2013). According to many researchers, compulsions in OCD constitute excessive attempts to gain control over threats and reduce anxiety (Carr, 1974; McFall and Wollersheim, 1979; Foa and Kozak, 1986). While some authors posit that these excessive control attempts are used to compensate for a lack of perceived control over life events (McLaren and Crowe, 2003; Moulding and Kyrios, 2006), others have suggested the very opposite, that these efforts to gain control reflect the OCD patient’s belief that they have an exaggerated sense of control or “power,” a factor thought to contribute to heightened responsibility cognitions (Salkovskis, 1996). Despite considerable discourse on the topic, there is little empirical data regarding control beliefs in OCD patients.

In one study, however, Reuven-Magril et al. (2008) employed a control estimation task in which participants attempted to decrease the duration that neutral and aversive images were displayed on the screen by using a sequence of button-presses. Participants were unaware that there was in fact no relationship between their button-press patterns and the duration that the image was presented. They found that obsessive–compulsive symptoms were related to an increased self-reported sense of control over the duration of the image and a decrease in key press variability. As the task employed in this study was novel, we sought to test if this effect was replicable using a task that conforms to the previous (and vast) literature on the illusion of control phenomenon.

In 1979, Alloy and Abramson reported that, in situations where there is no relationship between actions and outcomes, but the density of reinforcement is high, healthy individuals experience an optimism bias called the “illusion of control.” That is, they inappropriately infer contingency on the basis of how often reinforcing outcomes are presented to them. In their seminal report, Alloy and Abramson found that individuals suffering from depression do not succumb to the illusion of control bias. This finding was termed “depressive realism,” and has seen multiple replications (Alloy et al., 1981; Martin et al., 1984; Benassi and Mahler, 1985; Vazquez, 1987; Presson and Benassi, 2003; Msetfi et al., 2005, 2007). In this classic paradigm, participants are presented with an unlit light bulb and a button. Although there are many variations, subjects are typically informed that they can press or not press the button on a given trial and should try and figure out how much control they have over the light bulb. In the present study, we used the basic specifications of this paradigm, wherein outcomes were binary (successes or failures), there was no contingency between responses and light bulb illumination, and the density of outcome presentation (light bulb illumination) was experimentally manipulated using a block design.

The main differences between the paradigm employed by Reuven-Magril et al. (2008) and the classic paradigm, which we employed in the present study are as follows: (i) key press combinations are utilized in the Reuven-Magril et al.’s (2008) study rather than binary response vs. no-response options, (ii) these authors also used a gradual change in spurious reinforcement rather than a blocked design where reinforcement density is held constant within blocks, and (iii) they used continuous reinforcers, i.e., a reduction in the duration of a video, rather than binary outcomes of reward vs. no-reward (i.e., light bulb illuminates or does not).

### HYPOTHESIS

Obsessive–compulsive disorder patients will show an exaggerate illusion of control on the illusion of control task, in line with the results of a recent study (Reuven-Magril et al., 2008) employing a novel methodology for assessing control estimation. We did not predict any differences in terms of trial-by-trial behavior (number of responses or variability), as response options on this task are binary and subjects are explicitly encouraged to sample equally across the “response” and “no-response” options.

## MATERIALS AND METHODS

### PARTICIPANTS

This study was approved by the Cambridgeshire 2 Research Ethics Committee (10/H0308/27). Twenty-six OCD patients (15 females) and 26 age-matched comparison subjects (15 females) completed the experiment. OCD patients were screened by a psychiatrist using an extended clinical interview to ensure that they met the DSM-V criteria for OCD (APA, 2013), had severity scores exceeding 12 on the Yale–Brown Obsessive Compulsive Scale (YBOCS; Goodman et al., 1989), and had no co-morbid psychiatric disorders. We excluded OCD patients for whom hoarding was the primary complaint. Controls were recruited from the local community via advertisement and given the same study description as the patient group. Exclusion criteria for all participants were substance dependence and depression scores exceeding 16 on the Montgomery–Åsberg Depression Rating Scale (MADRS; Montgomery and Asberg, 1979). OCD patients had a mean YBOCS score of 22.58 (SD = 5.25), with mean values of 10.77 (SD = 3.82) and 11.77 (SD = 2.9) on the obsessions and compulsions subscales, respectively. As is characteristic of this population, OCD patients reported higher levels of depressive symptoms (though well below clinical threshold), anxiety, and personal responsibility (**Table 1**). OCD patients had spent significantly fewer years in education, but there were no differences in verbal IQ skills assessed using the National Adult Reading Test (NART; Nelson, 1982; **Table 1**). Eighteen patients were medicated [8 unmedicated; 1 tricyclic anti-depressant clomipramine; 1 antidepressant agomelatine; 10 SSRI (selective serotonin reuptake inhibitor) only; 4 SSRI + antipsychotic; 1 SSRI + lithium bicarbonate; 1 SSRI + beta-blocker propranolol]. Participants completed two additional tests in this battery, in a fully counterbalanced order. The results of which are published elsewhere (Gillan et al., 2013b), or in preparation. The entire session took approximately 2 h.

**Table 1:** Group demographics.

	Comparison subjects	OCD	*F*-value	df	*p*-Value
Age	40.38 (13.69)	42.5 (13.7)	<1	1,50	0.58
NART (errors)	13.96 (7.32)	14.92 (7.08)	<1	1,50	0.632
Education (year)	16.57 (1.77)	15.19 (2.49)	5.32	1,50	0.025
MADRS	1.15 (3.11)	6.69 (3.69)	34.322	1,50	<0.001
OCI-r	9.19 (8.29)	32.81 (11.14)	75.205	1,50	<0.001
STAI-state	30.54 (5.75)	44.19 (8.9)	45.11	1,50	<0.001
STAI-trait	33.54 (8.25)	60.04 (8.49)	130.29	1,50	<0.001
RAS	2.54 (0.746)	3.916 (1.13)	25.979	1,49	<0.001

*Mean scores and standard deviations (in parentheses). NART, National Adult Reading Test; OCI-r, Obsessive–Compulsive Inventory-revised; STAI, State-Trait Anxiety Inventory; RAS, Responsibility Attitude Scale.*

### PROCEDURE

Subjects were told that their goal was to illuminate a light bulb as often as possible by pressing or not pressing the spacebar. Subjects were informed that they also had to determine the contingency between their actions and the illumination of the light bulb. Each trial began with a display of an unlit light bulb. Participants decided whether or not to respond within 1.5 s. At the end of each section, subjects rated the degree of control they believed they had over the light bulb on a visual analog scale (VAS) ranging from 0 (“no control”) to 100 (“complete control”).

**FIGURE 1:**
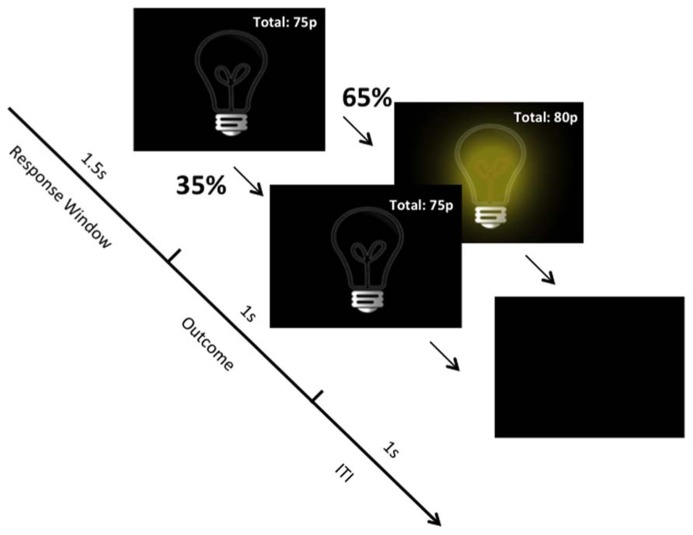
**Task structure.** Example from the “High Density/Gain” block. Independent of responding, on 65% of trials the light bulb illuminates and on 35% it does not illuminate. On trials where the light bulb illuminates, 5p is added to subjects’ total winnings.

Each block comprised 40 trials. There was never any contingency between button presses and the illumination of the light bulb. In two blocks, there was a high rate of non-contingent outcome presentation, where the light bulb illuminated on 65% of trials regardless of responding (**Figure 1**). In the two low reinforcement blocks, the rate of non-contingent outcome presentation was 35%. Reuven-Magril et al. (2008) found that differences associated with obsessive–compulsive symptoms are greatest when subjects had to control aversive (vs. neutral) images. Therefore, we included a valence factor wherein, in one of each of the high and low blocks, subjects were told that their goal was to avoid losing money, which started at £3.00. In the remaining two high and low blocks, subjects’ goal was to earn money, starting with £0. Gains and losses of 5p were instantly added to or subtracted from subjects’ total, which was presented in the top right corner of the screen. Outcome density was manipulated within, rather than between, subjects in the present study to increase statistical power. The four blocks were presented in a counterbalanced order across participants using a Latin square and subjects were informed that: “each of the four stages is independent of one another and therefore different rules may apply.” The experimenter was present during the experiment. Finally, we collected self-report questionnaire data on the Responsibility Attitude Scale (RAS; Salkovskis et al., 2000), Obsessive–Compulsive Inventory-revised (OCI-r; Foa et al., 2002), and the State-Trait Anxiety Inventory (STAI; Spielberger et al., 1983). Data from one subject on the RAS were lost due to a technical error.

### TASK INSTRUCTIONS

The following instructions were presented to participants on screen prior to beginning the experiment:

In this game, your task is to illuminate a light bulb as often as possible by pressing or not pressing the spacebar. One each trial, an unlit light bulb will appear on the screen and you will have the chance to do something:

(1) You may press the spacebar.
(2) You may not press the spacebar.

On each trial, the unlit light bulb will appear for 1.5 s. During this time you will have the chance to press the spacebar or not press the spacebar. A button press consists of pressing the spacebar once and only once during this 1.5 s interval. If you do not press the spacebar within this interval, it will be registered as a no press response. Therefore, there are four possibilities of what might happen on a given trial:

(1) You make a button press and the light bulb illuminates.
(2) You do not make a button press and the light bulb illuminates.
(3) You make a button press and the light bulb does not illuminate.
(4) You do not make a button press and the light bulb does not illuminate.

You will not be told anything about the relationship between pressing or not pressing the spacebar and the illumination of the light bulb. You must figure this out for yourself in each of the four stages of this experiment. Therefore, it is in your interest to press on some trials, but not on others, so that you know what happens when you don’t press as well as when you do press.

You can win some real money in this experiment! In some stages, every time the light bulb illuminates, you will win 5 pence. On others, every time the light bulb does not illuminate, you lose 5p. Whatever amount you win will be added to your check at the end of today’s testing session. Therefore on each of the four stages, it is in your interest to try and get the light to illuminate as often as possible. At the end of each of the four stages you will be asked to rate the degree of control you think you have had over the illumination of the light bulb.

Having **NO control** means that whether or not the light bulb illuminates has nothing to do with what you DO or do NOT DO. In other words, the illumination of the light bulb was totally determined randomly or by chance, rather than determined by your choice of responses, either pressing or not pressing. Having an **intermediate level of control**, means that your choice of responses, either pressing or not pressing influenced the illumination of the light bulb to some extent even though it did not completely determine whether or not it illuminated. Having **complete control** means that the illumination of the light bulb was completely determined by your choice of responses, either pressing or not pressing.

This definition of control was provided to subjects again, prior to making control ratings at the end of each of the experimental blocks. Prior to win and loss blocks (respectively), the instructions read:

“In this block, every time the light bulb illuminates, you will win 5p. Every time it does not illuminate, your total will not change”.

“In this block, every time the light bulb does not illuminate, you will lose 5p. Every time it illuminates, your total will not change”.

### QUESTIONNAIRES

Self-report questionnaire data collected were from the RAS (Salkovskis et al., 2000), OCI-r (Foa et al., 2002), and the STAI (Spielberger et al., 1983). The RAS is a 26-item self-report questionnaire that assesses responsibility cognitions. It was designed specifically to assess these cognitions in relation to obsessional symptoms (e.g., “many of my past actions have been intended to prevent harm to others”). The RAS has high test–retest reliability (*r* = 0.94), internal consistency (Cronbach’s α = 0.92) and can distinguish OCD patients from anxious controls (*p* < 0.001; Salkovskis et al., 2000). Data from one subject on the RAS were lost due to a technical error. The OCI-r is 18-item self-report questionnaire, which quantifies the severity of OCD symptoms across six symptom subscales (washing, checking, ordering, obsessing, hoarding, and neutralizing). The scale has good convergent validity with the clinician-administered YBOCS (*r* = 0.41; Abramowitz and Deacon, 2006) and adequate test–retest reliability (*r* = 0.7; Hajcak et al., 2004). The STAI is a 40-item self-report questionnaire that divides anxiety into “state” and “trait” phenomena. State anxiety refers to symptoms that are present at the time of testing (e.g., “I feel nervous”), while trait anxiety refers to anxiety that is generally present, most of the time (e.g., “I am a steady person”). The STAI has high test–retest reliability (median trait: *r* = 0.88, median state: *r* = 0.68) and internal consistency (median trait: α = 0.90, median state: α = 0.92; Barnes et al., 2002). We also conducted a test of reading ability using the NART (Nelson, 1982), as a proxy for intelligence. This test was designed to be relatively insensitive to cognitive decline, for example, associated with dementia, and is therefore often used a measure of premorbid intelligence in general.

### DATA ANALYSIS

Data were analyzed in SPSS using ANOVA, and Pearson’s correlations. For dependent measures control estimation, number of responses made, and mean reaction time (RT), mixed ANOVAs with Group (OCD, Comparison Subjects), Outcome Density (High, Low), and Valence (Gain, Loss) were computed.

## RESULTS

### ESTIMATION OF CONTROL

**FIGURE 2:**
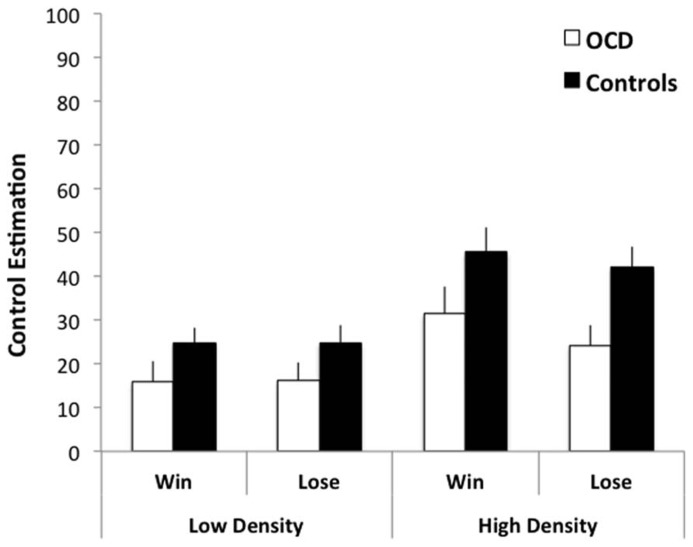
**Control estimations by OCD patients and comparison subjects during a zero contingency task.** OCD patients’ estimates were overall more realistic than comparison subjects independent of the outcome density (high-65%/low-35%) and valence (gain +5p/loss -5p) conditions. Error bars represent standard error of the mean.

Obsessive–compulsive disorder patients made more accurate control judgments than comparison subjects, evidenced by a significant main effect of group, *F*(1,50) = 5.148, *p* = 0.028, $\eta_{p}^{2}$ = 0.093, with comparison participants reporting greater levels of perceived control (*M* = 34%, SD = 16.6) than OCD patients (*M* = 22%, SD = 22.18; **Figure 2**). In line with the existing literature on the illusion of control, there was a highly significant main effect of outcome density (high, low), *F*(1,50) = 41.438, *p* < 0.001, $\eta_{p}^{2}$ = 0.453, with subjects reporting greater control in the high (*M* = 36%, SD = 26.06) relative to the low (*M* = 20%, SD = 18.84) condition. There was no effect of valence (gain, loss) on control estimation, *F*(1,50) = 1.133, *p* = 0.232, $\eta_{p}^{2}$ = 0.022 and no significant interactions between group and valence, *F* < 1, group and outcome density, *F*(1,50) = 2.277, *p* = 0.138, $\eta_{p}^{2}$ = 0.044, or outcome density and valence, *F*(1,50) = 1.263, *p* = 0.267, $\eta_{p}^{2}$ = 0.025.

Control estimations in the OCD group did not correlate with depression scores on the MADRS, or with measures of OCD symptom severity, the YBOCS and OCI-r, all non-significant, *r* < ∣0.33∣ . There was a significant positive correlation between responsibility attitude scores and overall control estimations in the OCD group, *r*(26) = 0.391, *p* = 0.048, such that the greater the extent of endorsing responsibility attitudes the higher the reported degree of control over the light bulb. This correlation, however, was not evident in the comparison subjects, *r*(25) = 0.193, *p* = 0.355, and did not survive correction for multiple comparisons.

### ANALYSIS OF COVARIANCE

The main effect of group remained significant when MADRS depression scores were entered as a covariate in the ANOVA, *F*(1,49) = 4.344, *p* = 0.042, $\eta_{p}^{2}$ = 0.081, suggesting that depression symptoms, which have been historically linked to control estimation bias, did not account for this effect. We repeated our analysis including years in education as a covariate, as this also differed significantly between groups. While years in education did not significantly affect control estimation ratings, *F*(1,49) = 2.414, *p* = 0.127, $\eta_{p}^{2}$ = 0.047, its inclusion as a covariate in our analysis reduced our main effect of group on control estimation to the marginal range: *F*(1,49) = 2.909, *p* = 0.094, $\eta_{p}^{2}$ = 0.056, suggesting that the difference in years in education observed across groups was contributing somewhat to our main effect of control estimation. However, analysis of covariance is particularly sensitive to outliers in the dependent variable. When we removed the control estimation ratings from one patient (>2 SDs from population mean) in the OCD group, our main effect of group was significant, *F*(1,48) = 6.129, *p* = 0.017, $\eta_{p}^{2}$ = 0.113, while the effect of education was unrelated to control estimation, *F*(1,48) = 1.659, *p* = 0.204, $\eta_{p}^{2}$ = 0.033.

### BEHAVIOR

There were no differences between groups in overall RTs, *F* < 1. Additionally, there were no main effects of outcome density, *F* < 1, or valence, *F*(1,50) = 2.219, *p* = 0.143, $\eta_{p}^{2}$ = 0.043, on RTs, nor were there significant interactions between group and density, group and valence, or density and valence, all *F* < 1. The three-way interaction between group, valence, and density was not significant, although in the marginal range, *F*(1,50) = 3.030, *p* = 0.088, $\eta_{p}^{2}$ = 0.057. There were no significant group differences in the number of responses performed (OCD: *M* = 22.28, SD = 4.2; Comparison Subjects: *M* = 21.8, SD = 5.72). Subjects responded overall on just over 50% of trials, consistent with their instructions. Additionally, there were no effects of outcome density or valence on the number of responses performed, all *F* < 1. The three-way interaction between density, valence, and group was also non-significant, *F*(1,50) = 1.312, *p* = 0.257, $\eta_{p}^{2}$ = 0.026.

## DISCUSSION

Using an illusion of control procedure that has been extensively studied in the literature (Alloy and Abramson, 1979), we observed that OCD patients report lower (and more accurate) estimations of control than healthy comparison subjects. Unlike the depressive realism phenomenon, lower estimates in the OCD group were not confined to the high outcome density blocks; rather OCD patients’ control estimates were consistently lower than comparison subjects regardless of density or valence. These data are inconsistent with those published by Reuven-Magril et al. (2008), yet not with their interpretation of their results. These authors, and others, suggest that OCD patients have a reduced sense of control and to cope, they use behavior (such as repeating key-press patterns) to compensate for this, instilling an illusory sense of control (Frost et al., 1993; Keinan, 1994; Zebb and Moore, 2003; Moulding et al., 2007; Reuven-Magril et al., 2008). Our data cannot speak directly to this hypothesis, but they provide tentative support for one of its tenets, that OCD patients have a reduced sense of control when behavior is held constant across individuals in terms of response vigor and pattern variability. Further research is needed to test whether behavior is a determinant of perceived control in OCD. Although, OCD patients were more accurate than controls at judging the absence of contingency in the present experiment, it remains to be seen if this in fact reflects improved accuracy or rather a flattening of contingency experience overall. The present study was not designed to parse this distinction. If the latter holds true, these findings might converge with more recent neurocognitive assessments that have identified a disturbance in action–outcome contingency learning in OCD (Gillan et al., 2011, 2013a). One possibility is that the difficulties linking actions and outcomes evident in OCD result in a flattened overall contingency experience, which may leave patients vulnerable to forming habits (Gillan et al., 2013b). However, this awaits direct test in an experimental setting where true contingency exists between action and outcome.

There was no evidence for a relationship between control estimation and depressive symptoms. This was expected, given that previous studies have shown that the depressive realism phenomenon breaks down when the experimenter is present while subjects carry out the task (Benassi and Mahler, 1985). There are three main limitations to this study. Firstly, we did not have a positive comparison group such as generalized anxiety disorder (GAD) patients, to delineate whether this effect is specific to OCD or a common feature of anxiety disorders. Secondly, the majority of OCD patients were receiving psychotropic medication, predominantly SSRIs. We did not have sufficient power to compare medicated and un-medicated patients in the present study; future studies should investigate if these medications have an influence on control estimation in this patient population. Finally, our groups differed significantly in the number of years spent in education. While this did not affect our results, there was a trend toward less time in education being predictive to lower estimates of control. This warrants further investigation.

To summarize, we did not replicate the findings of Reuven-Magril et al. (2008) who found that OCD patients had an exaggerated sense of control over external events. Using a more traditional illusion of control paradigm (Alloy and Abramson, 1979), we found evidence in the opposing direction, suggesting that OCD patients have a reduced sense of control. The most parsimonious explanation for this disparity is the sensitivity of control estimation to changes to task parameters. For example, healthy individuals typically experience an illusory sense of control only when there is (i) no contingency between actions and subsequent outcomes (Alloy and Abramson, 1979; Vazquez, 1987), (ii) the density of reinforcement is high (Alloy and Abramson, 1979; Benassi and Mahler, 1985; Vazquez, 1987; Presson and Benassi, 2003; Msetfi et al., 2005), (iii) outcomes are of a positive valence (Alloy and Abramson, 1979), (iv) inter-trial intervals (ITIs) are sufficiently long (Msetfi et al., 2005, 2007), and (v) an observer is not present during the experiment (Benassi and Mahler, 1985). Some have suggested that this volatility is indicative of control estimation being a decision-process rather than a perceptual one (Allan, 2002), and that the illusion of control task may not be an accurate measurement of sensitivity to contingency, but rather also reflects an individual’s willingness to predict that an outcome will occur, or in other words “say yes” (Allan et al., 2007). Future research is needed to assess this possibility and to ascertain whether this paradigm can provide meaningful clinical insight, given its well-documented fragility.

## Conflict of Interest Statement

The authors declare that the research was conducted in the absence of any commercial or financial relationships that could be construed as a potential conflict of interest.
